# Neo-Omphaloplasty within a Vertical Scar: The Double Trapezium Flap Technique

**DOI:** 10.3390/jcm13195659

**Published:** 2024-09-24

**Authors:** Thomas Holzbach, Katarina Danuser, Christine Sophie Hagen, Denis Ehrl, Sebastian Leitsch

**Affiliations:** 1 Department of Hand and Plastic Surgery, Thurgau Hospital Group, 8500 Frauenfeld, Switzerland; katarina.danuser@stgag.ch (K.D.); sebastian.leitsch@stgag.ch (S.L.); 2 Divison of Hand, Plastic and Aesthetic Surgery, University Hospital LMU Munich, 80336 Munich, Germany; christine_sophie.hagen@med.uni-muenchen.de; 3 Department of Plastic, Reconstructive and Hand Surgery, Center for Severe Burn Injuries, Klinikum Nürnberg, Paracelsus Medical University, 90431 Nuremberg, Germany; denis.ehrl@klinikum-nuernberg.de

**Keywords:** omphaloplasty, neo-omphaloplasty, umbilicoplasty, neo-umbilicoplasty, umbiliconeoplasty, abdominal reconstruction, indocyanine green fluoroscopy

## Abstract

**Background:** Various techniques for neo-omphaloplasty (or umbilicoplasty/umbiliconeoplasty) have been established in recent decades. However, when the omphaloplasty must be integrated into a vertical scar, most of these techniques are unsuitable. **Method:** We established a technique comprising two “cross-border” trapezium flaps that come together in a key-lock fashion to be applicable for umbilical reconstruction in vertical scars. Between 2020 and 2023, we performed the double trapezium flap technique in 11 patients requiring abdominal wall correction due to previous operations resulting in the loss of the original navel and a vertical midline scar. The follow-up period was 12 months. **Results:** We encountered two minor wound healing disorders not involving the omphaloplasty. One patient experienced a more severe wound healing complication involving the vertical scar and the lower flap of the neo-umbilicus. No cases of umbilical flattening or detachment of the anchorage stitches were detected. Patients ranked the aesthetic outcomes as “excellent” (*n* = 9) or “good” (*n* = 2). Physicians ranked the results as “excellent” (*n* = 7), “good” (*n* = 4), and “average” (*n* = 1). **Conclusions:** For the selected patients, this technique appears to be a good and reliable option to create a natural looking neo-umbilicus, creating sufficient umbilical depth with minimal scarring. While a study population of 11 patients is hardly enough to endorse a new technique, appropriate cases are comparatively rare and very specific.

## 1. Introduction

The umbilicus is our last remnant of our life in utero [1,2,3,4] and is essential to the aesthetic appearance of the abdomen [5]. While in most cases of abdominal wall reconstruction, abdominoplasty, or abdominal scar revision the existing umbilicus can be preserved and re-inserted, there are instances in which the existing umbilicus must be sacrificed. This may be due to umbilical or ventral hernia repair, pre-existing vertical scars that compromise the blood supply, omphalocele, or bladder exstrophy [1]. Here, it is essential to create a new umbilicus whenever possible to improve the aesthetic appearance of the abdomen.

The ideal shape and position of the umbilicus has been discussed extensively in the literature. The perfect umbilicus is found to be modest in size, T-shaped, or vertically shaped with a superior hood [5]. The ideal location of the umbilicus appears to be “low-riding”, meaning that the distance from the xiphoid process to the umbilicus is greater than the distance from the umbilicus to the pubic symphysis [6,7]. However, re-insertion of the umbilicus is planned based on anthropometric landmarks such as the xiphoid process, the lower limit of the vulvar cleft, the superior iliac spine, and the height of the waistline. Interestingly, Rohrich et al. found that, in most cases, the natural umbilicus is not located exactly in the midline [8].

Various neo-omphaloplasty (or umbilicoplasty/umbiliconeoplasty) techniques have been established in recent decades. The most common include the three flaps technique [9,10,11,12,13,14], the four flaps technique [15,16,17,18,19], the purse-string suture technique [20,21,22,23,24,25], the inverted C-V flap [26,27], the rabbit-head-shaped scar flap [28], the spiral rotational flap [29], and the dome procedure [30].

However, when the neo-omphaloplasty must be integrated into a vertical scar, most of these techniques cannot be used. The most common technique in these cases remains the “two lateral rectangular flaps technique,” first described by Sabatier in 1978 [31,32]. We used this technique as standard over many years but found it has its limitations regarding the aesthetic outcome. The most obvious downside is the insufficient width of the umbilical opening. This is due to the design—when the upper and lower borders are advanced, the midline closes and the two flaps form an umbilicus, which is a slit rather than an oval. While the recommended packing of the reconstructed cavity forms a round-to-oval depression [32], the desired effect in many cases is only temporary, since the limitations of the design remain unaltered [33].

In 2005, Pfulg et al. presented a new technique consisting of a triangular skin flap within the elliptical skin excision over the umbilicus in a horizontal direction [34]. The problem is this technique requires a 6 cm wide flap of well-perfused abdominal skin to create the new umbilicus. Assuming the scar is in the midline, for even distribution of skin tension, one must plan a midline resection at least 12 cm wide at the level of the new umbilicus—disregarding the width of the scar that must be removed additionally. In most of our cases, we would not have had sufficient excess skin.

**Figure 1:**
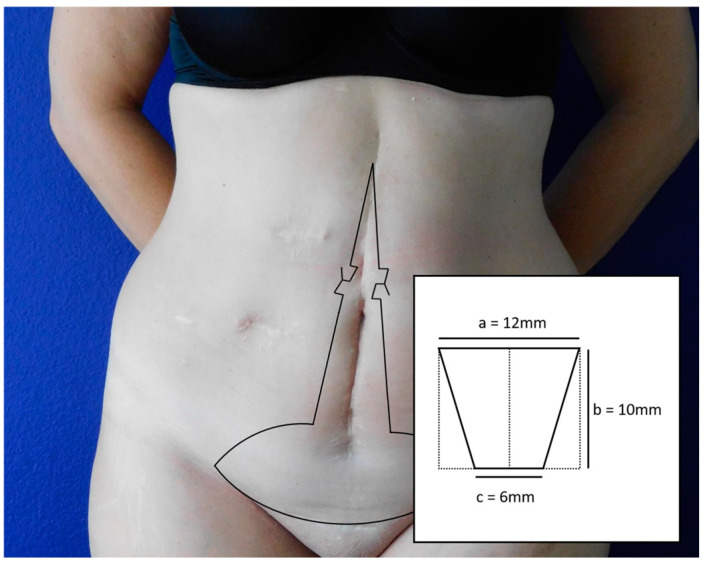
Surgical planning. The technique is based on the shape of two trapezium flaps, 12 mm in width and 10 mm in length each, interconnecting at the narrow base. The narrow base of each flap measures half of the width. Each flap is marked equatorially across the midline with opposite cut-outs and included in the midline resection The blood supply entering the flap is ensured over the non-dissected half distance of the wider base (6 mm), thus not exceeding the generally accepted length-to-width ratio of 1:2 for random-pattern flaps.

We developed a design that overcomes these limitations and allows the creation of an umbilicus with a defined width even when the upper and lower skin edges of the vertical incision are advanced. Furthermore, the design allows for an oval umbilicus with sufficient permanent depression and a natural appearance. To do this, we established a technique comprising two “cross-border” trapezium flaps with opposite cut-outs that come together in a key-lock fashion (Figure 1). This flap design was modeled after the H-wing neo-umbilicoplasty technique by Hoyos et al. [4] which we adapted to be applicable for umbilical reconstruction in vertical scars.

## 2. Material and Methods

We included all patients referred to our service for abdominal wall reconstruction or extensive scar correction of the abdomen with loss of the original umbilicus and a pre-existing vertical midline scar. We excluded all patients that were not eligible for direct umbilical reconstruction due to medical preconditions or severe obesity with a BMI > 40 kg/m^2^.

Between 2020 and 2023, we performed the double trapezium flap technique on 11 patients requiring abdominal wall correction due to previous operations resulting in the loss of the original navel and a vertical midline scar. The follow-up period was 12 months.

Postoperative complications were classified as Grade I to Grade V using the Clavien–Dindo classification of surgical complications [35]. Grade I corresponds to any deviation from the normal postoperative course including minor wound infections or prolonged wound healing requiring no need for further intervention, Grade II requires pharmacological treatment, Grade IIIa requires surgical intervention without general anesthesia, and Grade IIIb requires surgical intervention under general anesthesia. Grade IV corresponds to life-threatening complications requiring IC/ICU management with either single organ dysfunction (Grade IVa) or multiorgan dysfunction (Grade IVb), while Grade V represents the death of the patient.

The results were assessed by both physicians and patients after 12 months. Three board-certified plastic surgeons not involved in the initial surgery analyzed anonymized standard postoperative photographs taken from two angles (full frontal, 45° oblique) 12 months after surgery. The results were graded as “excellent”, “good”, “average”, or “poor”. When grading was heterogeneous, a majority decision was reached. Patients were asked to grade their results after their 12-month postoperative consultations.

The study was approved by our institutional research committee on 6 August 2020 (AZ: HPC2020-4). No additional approval was sought from the Kanthonal Ethics Board (EKOS Ostschweiz), for the following reasons: 1. Photographic analysis was performed retrospectively on anonymized patient data. 2. No additional examination of patients was planned/performed. 3. Our technique as a modification implies no change in the established treatment options.

This procedure is in accordance with swissethics (Swiss Association of Research Ethics Committees) and with the ethical standards presented in the 1964 Declaration of Helsinki and its later amendments (last updated 2013).

## 3. Surgical Technique

The technique is based on the shape of two trapezium flaps, 12 mm in width and 10 mm in length each, interconnecting at the narrow base. The narrow base of each flap measures half of the width. Each flap is marked equatorially across the midline with opposite cut-outs and included in the midline resection (Figure 1). The blood supply entering the flap is ensured over the non-dissected half distance of the wider base (6 mm), thus not exceeding the generally accepted length-to-width ratio of 1:2 for random-pattern flaps [36,37].

**Figure 2:**
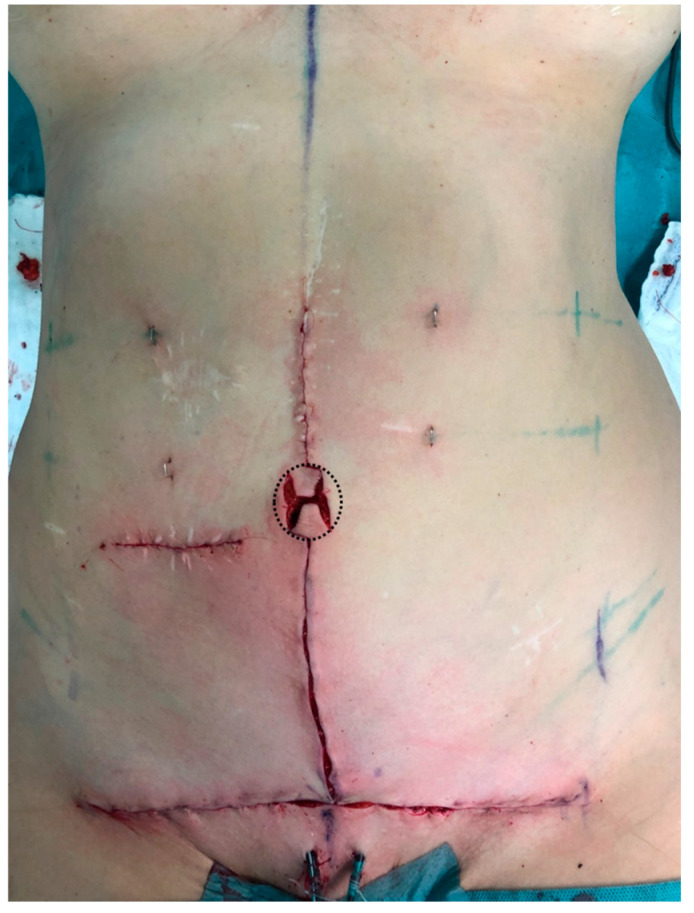
Surgical technique. Once the resection is completed, and the wound edges above and below the flaps are closed in the midline, the base of the flap is subdermally sutured into the cut-out. Subcutaneous defatting of the flaps and the lateral skin edges is performed, with attention to not harm the subdermal vascular plexus (dotted line).

**Figure 3:**
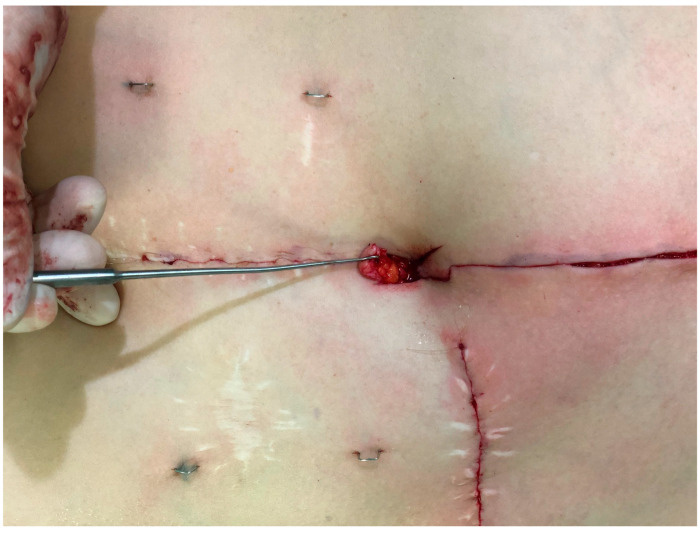
Surgical technique. The tip of the caudally based flap is sutured down to the fascia under slight upward traction. The lateral wound edges are separately sutured down on the same level. The superior flap serves as a hatch.

**Figure 4:**
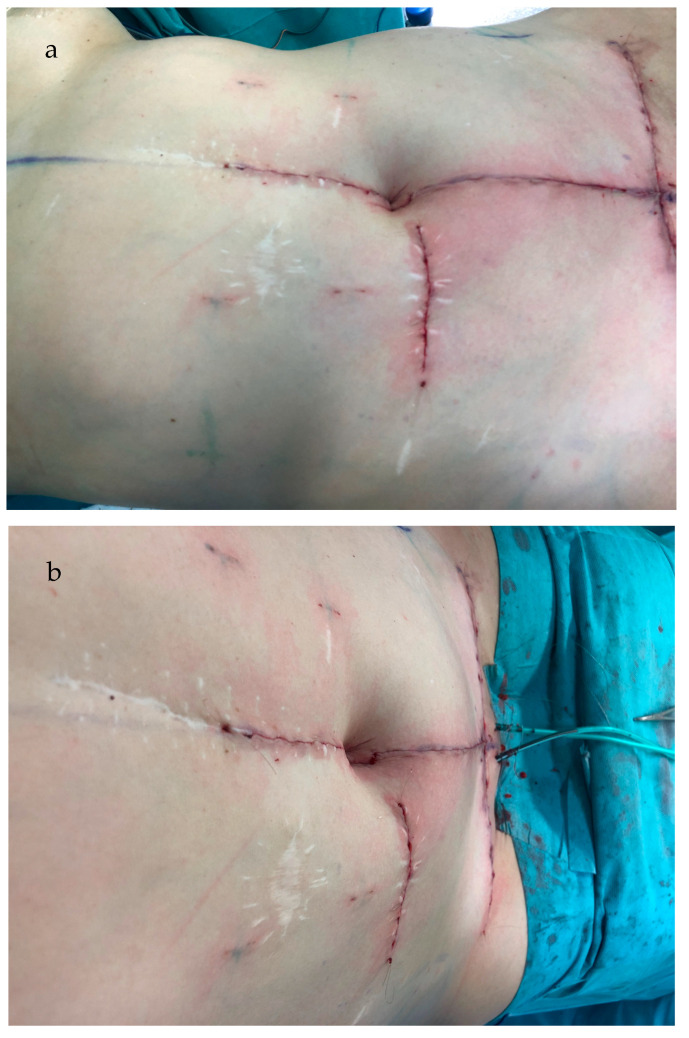
(**a**,**b**) Surgical technique. The superior flap is sutured down to the tip of the lower flap at the subdermal level without tension. Skin closure is completed using absorbable monofilament poliglecaprone 4-0 thread. The oblique lateral view (**a**) and oblique view from above (**b**) demonstrate sufficient umbilical depth and the desired oval umbilical shape.

Once the resection is completed, and the wound edges above and below the flaps are closed in the midline, the base of the flap is subdermally sutured into the cut-out, using absorbable braided polyglactin 4-0 thread (Vicryl^TM^, Ethicon^®^/Johnson & Johnson, New Brunswick, NJ, USA) (Figure 2). Then, subcutaneous defatting of the flaps and the lateral skin edges is performed, with attention to not harm the subdermal vascular plexus. The tip of the caudally based flap is then sutured down to the fascia under slight upward traction using absorbable braided polyglactin 2-0 threads at either side. The lateral wound edges are separately sutured down on the same level using 2-0 Vicryl^TM^ threads. The superior flap serves as a hatch (Figure 3) and is sutured in a final step down to the tip of the lower flap at the subdermal level without tension using 4-0 Vicryl^TM^. (Figure 4a,b). Skin closure is completed using absorbable monofilament poliglecaprone 4-0 thread (Monocryl^TM^, Ethicon^®^/Johnson & Johnson, New Brunswick, NJ, USA).

## 4. Indocyanine Green Fluoroscopy

**Figure 5:**
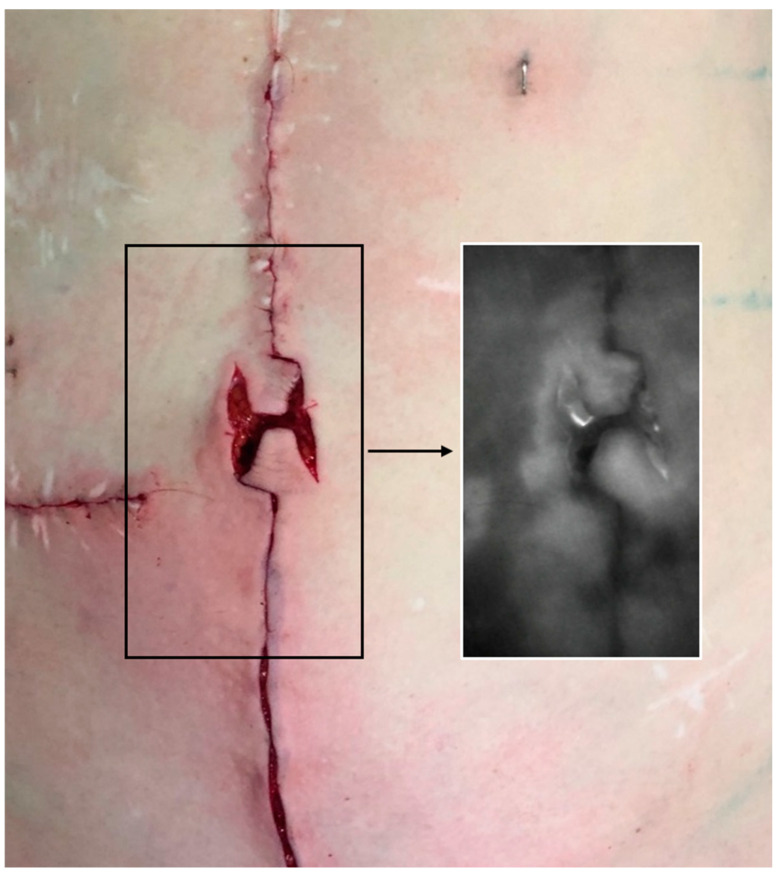
Indocyanine green fluoroscopy. We routinely performed indocyanine green (ICG) fluoroscopy. Analysis was conducted after closure of the midline and localized subcutaneous defatting. ICG fluoroscopy demonstrates sufficient perfusion of the flaps.

As is standard in our department when performing abdominal wall reconstruction with tissue resection, we routinely performed indocyanine green (ICG) fluoroscopy. Analysis was conducted after closure of the midline and localized subcutaneous defatting. Topographic perfusion analysis was performed after intravenous injection of 25 mg indocyanine green (ICG-Pulsion^®^ 5 mg/mL, PULSION Medical Systems SE, Feldkirchen, Germany), using the handheld IC-Flow^TM^ device (Diagnostic Green LLC, Farmington Hills, MI, USA) (Figure 5).

## 5. Results

**Figure 6:**
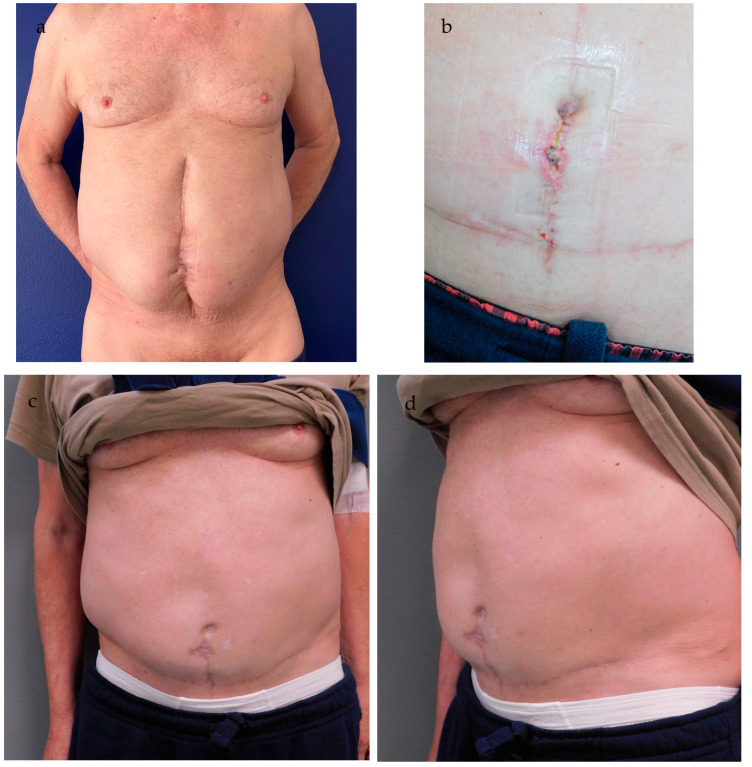
(**a,b**) Pre- early postoperative result. (**a**) 66-year-old patient with instable abdominal scarring and chronic fistula after multiple abdominal surgeries including recto-sigmoid resection, partial bladder resection, hernia repair, and multiple revisions after adhesive ileus of the small intestine. Pre-existing illnesses included diabetes Type 2, C2- and nicotine-abuse, and liver cirrhosis. (**b**) This patient experienced a Type IIIb wound healing complication involving the vertical scar and the lower flap of the neo-umbilicus. We opted for an early revision and decided to reconstruct the lower part of the umbilicus with a skin graft rather than waiting for secondary wound healing. Figure 6c,d show the postoperative result after 12 months, with the (**c**) full frontal and (**d**) 45° oblique view. The patient presented a superficial necrosis lateral to the vertical scar in the lower abdomen, 2.5 cm below the neo-umbilicus in the postoperative course. The neo-umbilicus itself healed without complications after skin grafting. The neo-umbilicus sustained sufficient depth and adequate shape. The patient rated the result as “good”, while the physicians rated the result as “average”. Retrospectively, in this case we should have opted for a simple skin grafting technique in the first place or decided against immediate umbilical reconstruction during abdominal wall reconstruction entirely.

We encountered three wound healing complications. Two were minor wound healing disorders (Clavien–Dindo Grade I) involving the T-scar, and the omphaloplasty was unaffected in both cases. One patient experienced a more severe wound healing complication involving the vertical scar and the lower flap of the neo-umbilicus (Clavien–Dindo Grade IIIb). The patient suffered from severe pre-existing illnesses, including ascites from liver cirrhosis, Diabetes Type 2, and C2-abuse and was a heavy smoker. We opted for an early revision and decided to reconstruct the lower part of the umbilicus with a skin graft from the groin rather than waiting for secondary wound healing (Figure 6a,b). Healing after revision was prolonged. The patient presented a superficial necrosis lateral to the vertical scar in the lower abdomen, 2.5 cm below the neo-umbilicus. The neo-umbilicus itself healed without complications after skin grafting (Figure 6c,d). We did not encounter any case of umbilical flattening or detachment of the anchorage stitches from the fascia.

The patients and physicians rated the aesthetic appearances differently after twelve months. The physicians ranked the appearance of the neo-umbilicus as “excellent” in seven cases, “good” in three cases, and “average” in one case. The patients rated the results as “excellent” in nine cases and “good” in two cases (Table 1). The physicians did not rate the results higher than the patient did in any case. However, we gathered additional free text information in selected cases. The one case that was ranked “average” by the physicians was the case of the Clavien–Dindo Grade IIIb wound healing complication that had to be treated with secondary skin grafting of the lower umbilical pole. The reasons for the “average” ranking were the “visible scarring” and the “noticeable difference of skin texture”. The corresponding patient commented that he considered it “good” that the “skingraft has a slightly different colour” compared to the rest of the abdomen. The other patient that considered the result to be “good” made the remark that her initial naval used to be more “crinkly” while the new one is “relatively“ smooth.

## 6. Discussion

**Figure 7:**
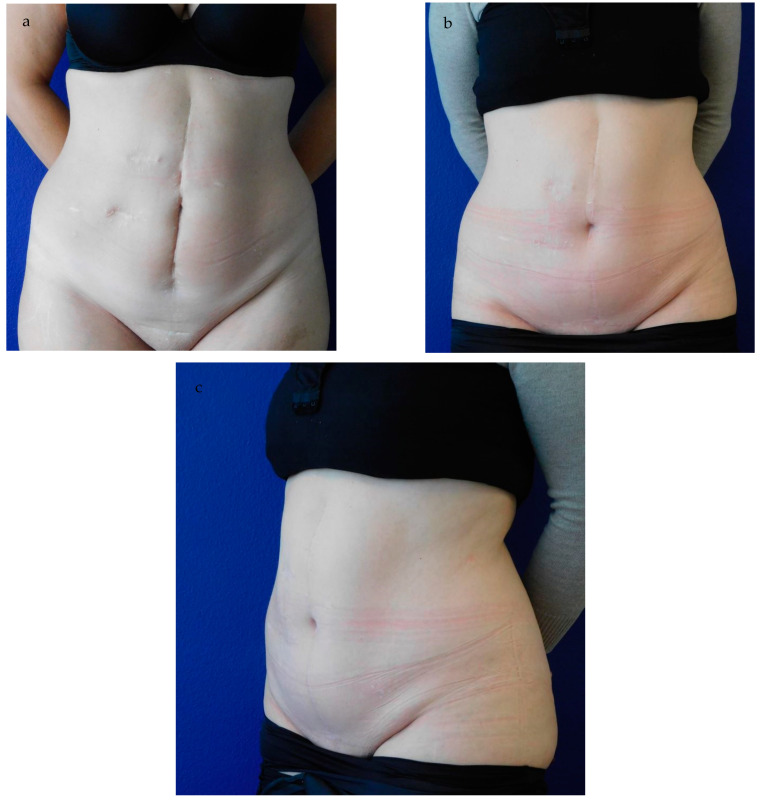
(**a**–**c**). Pre- and 12-month-postoperative result. (**a**) shows a 45-year-old patient with multiple scarring and loss of the umbilicus after repeated laparotomies after hemicolectomy, anastomotic leakage, intestine ischemia, and ileus. (**b**) presents the full frontal and (**c**) the 45° oblique view 12 months postoperatively. Both the patient and physicians rated the postoperative result as “excellent”. The neo-umbilicus sustained sufficient depth, an appealing shape, and minimal scarring.

Wherever there are multiple surgical techniques and modifications used concurrently over a long period of time, one must assume that the one perfect technique has not been established yet. Our technique certainly is not the answer to the limitations of the pre-existing techniques, but we present a relatively simple and safe approach that may be applicable in most cases. While this technique is feasible in all vertical midline scars or para-midline scars that can be converted into a midline scar, we recommend excluding patients with impaired microcirculation, diabetes mellitus, coagulation disorders, and ascites. For select patients, the double trapezium flap technique is a good and reliable option to create a natural-looking neo-umbilicus with sufficient depth and minimal scarring (Figure 7).

Most techniques are developed to form a neo-umbilicus after abdominoplasty, ventral hernia repair, omphalocele, or bladder exstrophy [1,9,10,11,12,13,14,15,16,17,18,19,20,21,22,23,24,25,26,27,28,29,30]. Here, the caseload is relatively high in certain centers. Cases in which the umbilicus must be reconstructed within a vertical scar are comparatively rare. First, we searched for a conventional umbilicoplasty technique that yielded favorable results after abdominoplasties or in cases without a midline scar present. We found the H-wing-neo-umbilicoplasty technique presented by Hoyos et al. to be advantageous over many others [4]. We gained some experience with this technique and tried to adapt it to incorporate a similar flap design into cases with a vertical midline scar. The flap design does not exceed the established length-to-width ratio of 2:1 for random pattern flaps, even though the blood supply over the remaining skin bridges is limited [36,37] When there are no contraindications, we routinely perform ICG laser fluoroscopy in abdominal wall reconstructions with multiple pre-existing scars. Therefore, we felt comfortable to progress with our modification, since we were able to monitor the perfusion of the trapezium flaps intraoperatively.

## 7. Limitations

Several limitations must be addressed. First, a study population of 11 patients is insufficient to endorse either a new technique or a relevant modification of an existing one. However, appropriate cases are comparatively rare and very specific. To the best of our knowledge, this is the largest case series for neo-omphaloplasties incorporated into midline scars. Still, more follow-up studies with a larger collective of patients are necessary to verify our promising results.

Second, this technique may not be ideal for every patient. We encountered one umbilical wound healing complication requiring surgical revision in a male patient with ascites from liver cirrhosis, diabetes mellitus type 2, and a history of nicotine and C2 abuse. Retrospectively, in this case, we should have opted for a simple skin grafting technique or decided against immediate umbilical reconstruction during abdominal wall reconstruction.

Finally, we cannot fully exclude a biased rating of the postoperative result by the patients. While we explicitly asked each patient to assess solely the appearance of the neo-umbilicus, we do not know if the general improvement of the appearance of the abdomen led to a more favorable rating of the umbilicus itself. In addition, when patients were pleased with their overall improvement and were asked to rank specific portions of the results, they may have rated the aesthetic appearance of their new navels benevolently.

## 8. Conclusions

For select patients, the double trapezium flap technique is a good and reliable option to create a natural-looking neo-umbilicus within a vertical scar. This technique creates sufficient neo-umbilical depth and is associated with minimal scarring. For patients with relevant comorbidities, impaired microcirculation, or coagulation disorders, we recommend using a simple skin grafting technique or refraining from umbilical reconstruction entirely.

## Figures and Tables

**Table 1:** Patient demographics.

	Patients (*n* = 11) Mean ± Std (Min, Max)	
Age at Surgery (y)	59.9 ± 11.5 (39, 75)	
Gender (f/m/d)	f = 7, m = 4	
Body Mass Index (kg/m^2^)	24.9 ± 2.3 (20.9, 29.4)	
Diabetes	*n* = 1	
Smoking	*n* = 3	
Complications [35] (Grade I-V)	Grade I: *n* = 2 * Grade IIIb: *n* = 1 **	
Result after 12 months (“excellent”, “good”, “average”, “poor”)	Physicians’ Assessment Excellent: 7/11 Good: 3/11 Average: 1/11	Patients’ Assessment Excellent: 9/11 Good: 2/11

* Wound dehiscence of T-scar, conservative treatment; umbilicoplasty unimpaired. ** Wound dehiscence including lower flap of umbilicoplasty, with surgical revision and skin grafting of lower umbilical pole under general anesthesia.

## Data Availability

Supporting data are available from the authors upon request.
